# Self-Monitoring with or Without Telemonitoring: Is a New Time for Diagnosis and Management Hypertension?

**DOI:** 10.36660/abc.20190701

**Published:** 2019-11

**Authors:** Cibele Isaac Saad Rodrigues

**Affiliations:** 1 Faculdade de Ciências Médicas e da Saúde - Departamento de Medicina - Área de Nefrologia, Sorocaba, SP - Brazil; 2 Pontifícia Universidade Católica de São Paulo (PUC-SP), São Paulo, SP - Brazil

**Keywords:** Cardiovascular Diseases, Telemedicine, Hypertension/prevention and control, White Coat Hypertension, Masked Hypertension, Hypertension Self-Monotoring

According to the 2017 American College of Cardiology (ACC)/American Heart Association (AHA) guideline for the prevention, detection, evaluation, and management of high blood pressure (BP) in adults, 46% of US adults have hypertension (HTN).^1^ In Brazil, because the levels of systolic and diastolic BP that define HTN^2^ differ from ACC/AHA Guideline, the percentage is about 25%, according to VIGITEL.^3^

HTN is an important public health issue and the major risk factor for heart disease, stroke, and chronic kidney disease. Heart disease and stroke are among the most prevalent and costly health problems worldwide, and controlling BP is the best effective intervention in life-saving, preventing deleterious comorbidities, but only about half of those with HTN have it under control even in developing countries.^1,2,4^

BP is one of the most important monitoring parameters in clinical medicine. Measuring BP properly is a crucial point in the diagnosis and management of HTN in clinical practice, in office settings or outside the office.^5^ Home BP monitoring with validate automatic devices become an alternative to Ambulatory Blood Pressure Measurement (ABPM), that is recommended as the preferred out-of-office BP method in the majority of guidelines^1,2,5,6^ because of its superior precision, unique ability to measure nocturnal BP identifying non-dippers and risers (reverse dipping), detects circadian variability and appears to correlate best with prognosis. In contrast to ABPM, HBPM is well-tolerated, more easily available, with lower cost, and also has a stronger association with renal and cardiovascular risk than office BP. Additionally, HBPM can give patients empowerment and a more active role in the management of their chronic disease, which is useful for monitoring the efficacy of antihypertensive treatment, thus improving BP control by diminishing medical therapeutic inertia. However, the scientific data at the moment recognized that HBPM, instead of being cost-effective and a good approach, is underutilized in the United States and other countries,^7^ and a few well-designed trials are available.

TASMINH4,^8^ a study conducts by National Institute for Health Research in United Kingdom, recently published in Lancet, found that self-monitoring, with or without telemonitoring, when used by general practitioners in primary care to titrate antihypertensive medication in individuals with uncontrolled BP, determined significantly lower systolic BP in intervention group than titration guided by office clinic readings. The results showed that self-monitoring with or without telemonitoring is beneficial in HTN management in primary care.

The Telescot^9^ is a Scottish trial that included seven studies on the implementation of telemonitoring in a primary care setting for the long-term management of HTN, diabetes and other chronic diseases. The results showed a high approval rate by patients, who found the new strategy empowering and useful. Physicians also considered telemonitoring encouragingly; however, some of them expressed criticism on software characteristics, which may denote obstacles to widespread implementation of telemonitoring in the routine of daily care.

In the study “Prevalence of Masked and White-Coat Hypertension in Pre-Hypertensive and Stage 1 Hypertensive Patients with the Use of TeleMRPA” in this issue, Barroso WKS et al.^10^ used the strategy of HBPM associated to telemedicine (TeleMRPA platform). The sample consisted of 1,273 participants, 58.1% were women, mean age was 52.4 ± 14.9 years, mean body mass index 28.4 ± 5.1 kg/m^2^. The casual in-office BP was higher than the HBPM (+7.6 mmHg for systolic and +5.2 mmHg for diastolic BP (p < 0.001). They found 558 (43.8%) normotensive individuals; 291 (22.9%) with sustained HTN; 145 (11.4%) with masked HTN (MH) and 279 (21.9%) with white-coat HTN (WCH), with a diagnostic error by casual BP in the total sample in 424 (33.3%) patients. In stage 1 hypertensive, the prevalence of WCH was 48.9%; in prehypertensive patients, the prevalence of MH was 20.6%. They concluded that MH and WCH have a high prevalence in the adult population, particularly in pre-hypertensive and stage 1 hypertensive patients and out-of-office BP measurements in these patients is particularly indicated to prevent misdiagnosing. In fact, it is an important result because prehypertension is a warning sign of developing long term HTN and at this stage, and even more at stage 1 HTN, they are both associated with target organ damage by increasing risk for cardiovascular and renal disease.^11^

As described above, telemonitoring is now successful used in primary care worldwide and we have to consider this strategy as a possibility to do our work in Brazil in terms of diagnosing and management of HTN. HBPM with or without telemonitoring can be used systematically in the daily practice as ABPM does to identify patients with white-coat HTN; masked HTN; sustained control, uncontrolled, resistant, or refractory HTN like the authors found in the analyzed paper.^1,2,5,6^

Nevertheless, it’s important to consider some limitations. It is a retrospective unblinded study that is capable of identifying a feasibility issue but not design specifically to extract data. The majority of the records was taken from patients that live in Northeast of Brazil and it’s a possible bias admitted for the authors. We don’t know how the patients comply with making the arterial pressure measurements in almost 30 different centers from nine states of the Brazilian geographic regions. It is a huge effort in training all patients and maintaining the devices well-calibrated and checked for accuracy periodically using a standard method with proper cuffs size and correctly positioned. Another patient-barrier is that HBPM requires literacy so that individuals are able to apply a standard measurement of BP using the same calibrated automatic oscillometric devices. Training of health professionals and patients is obligatory in all centers and is known that is time-consuming.^5^ Finally, we have other variables to consider like the presence of records signal artifact by movements or cardiac arrhythmia.

Is expected that in the future with an increasing use of digital medicine we will have new technologies like a next-generation BP monitoring devices, including smartphones and Bluetooth-enable telemonitoring, using cuffless and continuous BP monitoring systems that will be able to provide novel, accurate and validated solutions in clinical setting, improving outcomes of hypertensive patients.^12,13^ Maybe at this time, even ABPM will be part of the past.

An example of this new technology is the experimental watch-type prototype proposed by Woo et al.^14^ which uses a wearable device with a pressure sensor close to the radial artery, giving an accurate continuous BP measurement on a personal smartphone.

On the other hand, transmitted data from these home equipment and personal devices will create expectations of therapeutic decision making, if required. Another point to consider is that better levels of privacy and sensitive data security, integrity and safety control are essential, since flaws may occur with data sharing between patients and their health managers. In many countries, like in Brazil, we did not have clear technical, legal and ethical regulatory rules for e-health interventions and these are important tools to guide doctors in their clinical settings in an era of growing artificial intelligence.

The study of Barroso WKS et al.,^10^ brings us the results that out-of-office BP monitoring into Brazilian professional’s practice is an important complement to office HTN screening measures and is expected that a rigorous process of telemonitoring can break some barriers.

In conclusion, HBPM telemonitoring seems to be a promising patient management approach, particularly in cases of patients at high risk, that produces accurate and reliable data but there is a need to additional prospective randomized controlled trials of self-monitoring BP with and without telemonitoring compared with usual care and ABPM, with larger sample size and longer follow-up to confirm the results in literature about clinical effects on diagnosing, controlling and impact on morbidity and mortality of HTN patients. Cost-effectiveness of each strategy compared to another, impacts on services utilization, acceptance by professionals and patients are also essential for allowing scientific recommendations on the employment of these new tools in daily care for diagnosis and management HTN.
